# Detecting Hypoglycemia Incidents Reported in Patients’ Secure Messages: Using Cost-Sensitive Learning and Oversampling to Reduce Data Imbalance

**DOI:** 10.2196/11990

**Published:** 2019-03-11

**Authors:** Jinying Chen, John Lalor, Weisong Liu, Emily Druhl, Edgard Granillo, Varsha G Vimalananda, Hong Yu

**Affiliations:** 1 Department of Population and Quantitative Health Sciences University of Massachusetts Medical School Worcester, MA United States; 2 Bedford Veterans Affairs Medical Center Center for Healthcare Organization and Implementation Research Bedford, MA United States; 3 College of Information and Computer Sciences University of Massachusetts Amherst Amherst, MA United States; 4 Department of Computer Science University of Massachusetts Lowell Lowell, MA United States; 5 School of Medicine Boston University Boston, MA United States; 6 Department of Medicine University of Massachusetts Medical School Worcester, MA United States

**Keywords:** secure messaging, natural language processing, hypoglycemia, supervised machine learning, imbalanced data, adverse event detection, drug-related side effects and adverse reactions

## Abstract

**Background:**

Improper dosing of medications such as insulin can cause hypoglycemic episodes, which may lead to severe morbidity or even death. Although secure messaging was designed for exchanging nonurgent messages, patients sometimes report hypoglycemia events through secure messaging. Detecting these patient-reported adverse events may help alert clinical teams and enable early corrective actions to improve patient safety.

**Objective:**

We aimed to develop a natural language processing system, called HypoDetect (Hypoglycemia Detector), to automatically identify hypoglycemia incidents reported in patients’ secure messages.

**Methods:**

An expert in public health annotated 3000 secure message threads between patients with diabetes and US Department of Veterans Affairs clinical teams as containing patient-reported hypoglycemia incidents or not. A physician independently annotated 100 threads randomly selected from this dataset to determine interannotator agreement. We used this dataset to develop and evaluate HypoDetect. HypoDetect incorporates 3 machine learning algorithms widely used for text classification: linear support vector machines, random forest, and logistic regression. We explored different learning features, including new knowledge-driven features. Because only 114 (3.80%) messages were annotated as positive, we investigated cost-sensitive learning and oversampling methods to mitigate the challenge of imbalanced data.

**Results:**

The interannotator agreement was Cohen kappa=.976. Using cross-validation, logistic regression with cost-sensitive learning achieved the best performance (area under the receiver operating characteristic curve=0.954, sensitivity=0.693, specificity 0.974, F1 score=0.590). Cost-sensitive learning and the ensembled synthetic minority oversampling technique improved the sensitivity of the baseline systems substantially (by 0.123 to 0.728 absolute gains). Our results show that a variety of features contributed to the best performance of HypoDetect.

**Conclusions:**

Despite the challenge of data imbalance, HypoDetect achieved promising results for the task of detecting hypoglycemia incidents from secure messages. The system has a great potential to facilitate early detection and treatment of hypoglycemia.

## Introduction

### Significance and Background

Diabetes mellitus is a highly prevalent disease estimated to affect 425 million people worldwide with a cost of US $673 billion in 2015 [1]. Glycemic control is important for preventing long-term complications of diabetes. However, many factors, including improper dosing of antidiabetic medications such as insulin and sulfonylureas, can lead to hypoglycemic episodes, increasing the risk of severe morbidity or even death [2-5]. It is important to report these hypoglycemia incidents to clinical teams quickly, so that early corrective actions can be taken to improve patient safety.

Secure messaging is a popular functionality of patient portals [6-9] that has been increasingly used in recent years [10,11]. This technology allows for secure communication between patients and health care providers between episodic in-person clinic visits. The use of secure messaging has been associated with improved health care quality and outcomes [12-18]. Although secure messaging was designed for exchanging nonurgent messages (eg, clinical teams in the US Veterans Affairs’ [VA] health care system are allowed up to 3 days to respond to patients’ messages), patients sometimes use it to report urgent issues that need immediate attention, including adverse events [19-21]. It is important for health care providers to attend to these urgent issues early.

However, the volume of patient-provider secure messages can be huge. For example, the number of secure messages exchanged in the first quarter of 2018 at VA’s My Health*e*Vet [8,22] reached about 3.7 million [23]. It is beneficial and necessary to use automated methods to facilitate inspection of these data.

### Objective

This study aimed to develop HypoDetect (Hypoglycemia Detector)—to our knowledge, the first natural language processing (NLP) system to automatically identify hypoglycemia incidents from patients’ secure messages to facilitate timely responses. This task is challenging because, like other adverse events, hypoglycemia incidents are rare. In addition, patients report hypoglycemia in diversified, informal ways (detailed in the Results and Discussion sections). We addressed this challenge by using supervised learning methods with strategies to handle data imbalance and empirically evaluated our approach.

### Related Work

#### Natural Language Processing for Secure Message Classification

Previously, secure messages were typically analyzed by human experts [20,24], which is difficult to scale up.

Recent studies applied NLP methods to analyze secure messages to identify patients’ information needs [25-27]. Cronin and colleagues applied machine learning methods to classify patients’ information needs into 5 categories: clinical information, medical, logical, social, and other [25,26]. They found that random forest and logistic regression models and term frequency features were most effective for this task. Sulieman and colleagues extended that work by investigating new semantic and contextual features and deep learning models such as convolutional neural networks [27]. They found that convolutional neural networks with paragraph embeddings outperformed other models.

#### Learning From Imbalanced Data

Imbalanced data refers to datasets in which some classes have much fewer instances than others. Without treating data imbalance, automated systems often have poor recall (sensitivity) for the minority class [28], which will be a severe problem when the minority class is the target to predict.

Previous work in learning imbalanced clinical data focused on cancer screening and diabetes diagnosis [29-32]. For example, Zahirnia and colleagues adopted a hybrid cost-sensitive learning approach to predict diabetes [29]. Ramezankhani and colleagues showed that the synthetic minority oversampling technique (SMOTE) improved classifiers’ sensitivity, but not precision and F1 score, when predicting diabetes [31].

Blagus and Lusa empirically studied two methods for combining cross-validation and sampling techniques [32]. The first one divides the dataset into multiple folds and then samples the training set in each fold independently. The second method first samples the whole dataset and then divides the sampled dataset into multiple folds. Their results indicated that the first method is technically correct, especially for oversampling techniques including SMOTE. This is because oversampling on the whole dataset is likely to add similar or identical instances into both the training and test sets, causing an overestimation of classification performance.

## Methods

### Secure Messages

We collected 3000 secure message threads between patients with diabetes and VA clinical teams for this study. A secure message thread (abbreviated as *a thread* for convenience) refers to a single, entire thread of messages exchanged between a patient and his or her VA clinical providers. A message refers to a single instance of a communication in a thread. A thread includes 1 or more messages.

We conducted our data sampling process in 2 stages. In the first stage, we obtained a list of patient identification numbers from the VA data service for patients who had a diabetes outpatient visit (*International Classification of Diseases, Ninth Revision* [ICD-9] codes: 249.x and 250.x; *ICD Tenth Revision* [ICD-10] codes: E08-E13) between 2009 and 2017 and used these identification numbers to retrieve 2.3 million secure message threads from the VA’s Corporate Data Warehouse (Office of Information Technology, Department of Veterans Affairs, Washington, DC, USA).

In the second stage, we sampled our evaluation set from the 2.3 million threads obtained in stage 1. We first randomly sampled 1000 threads. An expert in public health annotated those threads and found that only 1 contained a hypoglycemia incident. We therefore used an enrichment approach to sample 2 additional sets of threads. Specifically, we used 2 rule-based methods to improve the recall of positive examples. Both methods constrained their sampling to outpatient visits that had diagnosis codes related to hypoglycemia (ICD-9 codes: 251.0, 251.1, 251.2; ICD-10 codes: E16.1, E16.2) and to the secure message threads sent within 30 days before or after those outpatient visits. The first method randomly sampled 1000 threads that contained at least one of the following keywords: blur, confused, dizzy, headache, hungry, pale, shake, sleepy, sweat, weak, dose, drop, and down. We selected these keywords based on information from the “Hypoglycemia” webpage posted on the US National Institute of Diabetes and Digestive and Kidney Diseases’ website [33]. The second method randomly sampled 1000 threads that contained the keyword sugar and at least one of the keywords used in the first method. We searched keywords by using a fuzzy match algorithm written in Transact-SQL that could retrieve inflectional variants of a keyword. We ensured that the 3 sets of threads had no duplicates and combined them into a single set (3000 threads in total) for this study.

### Annotation

An expert in public health who has worked in the civilian and military health care fields for 18 years annotated each thread as containing a hypoglycemia incident (positive) or not (negative).

We created a simple annotation guideline based on the American Diabetes Association’s standard [34] and Miller et al [2] (see Multimedia Appendix 1). We deemed a message to be positive if it (1) mentioned a blood glucose level <70 mg/dL (<3.9 mmol/L) [34], or (2) described typical hypoglycemia symptoms [2] that could not be contextually attributed to other possible causes (eg, high blood sugar and low blood pressure). We annotated a message thread as positive if it contained at least one positive patient message.

Multimedia Appendix 1 shows excerpts from several deidentified positive and negative messages in our dataset. It is worth noting that we judged as positive any messages that did not report blood glucose levels or reported borderline values but included typical symptoms in a context where hypoglycemia was likely to have occurred (eg, skipping a meal, taking diabetes medication, and then feeling lightheaded and sweaty). Examples 2 and 3 in the first table in Multimedia Appendix 1 fall into this category. We judged as negative the messages that were too vague (ie, lacking clear context) to determine whether an incident of hypoglycemia had occurred. Examples 2 and 4 in the second table in Multimedia Appendix 1 fall into this category.

We further asked a physician who specializes in family medicine to annotate 100 threads from these data independently. The 100 threads combined 2 sets of data. The first set contained 50 threads randomly selected from the 3000 threads, with 5 positive and 45 negative threads. The second set contained 25 randomly selected positive threads and 25 randomly selected negative threads.

### Training and Evaluation Data

We trained and evaluated HypoDetect by 10-fold cross-validation (detailed in Experimental Settings). We found that a secure message thread frequently contained 2 or more secure messages from patients and providers, with the first message being from the patient to raise questions or report problems. Because our goal was to develop a system to facilitate timely response to patient-reported hypoglycemia, we expected the system to make a prediction right after seeing the first patient message. In addition, we found that patients almost always reported hypoglycemia incidents in the first message of a thread. Therefore, we used the first message from each thread for our experiments.

This treatment also helped us to regularize the data. For example, it reduced the length variation of training and test examples, a factor that may affect the effectiveness of frequency-based features such as term frequency-inverse document frequency (TF-IDF). In addition, it helped the system focus on text regions where patients reported problems (including hypoglycemia events) and reduced distractive signals elsewhere.

### The HypoDetect System

#### System Overview

**Figure 1:**
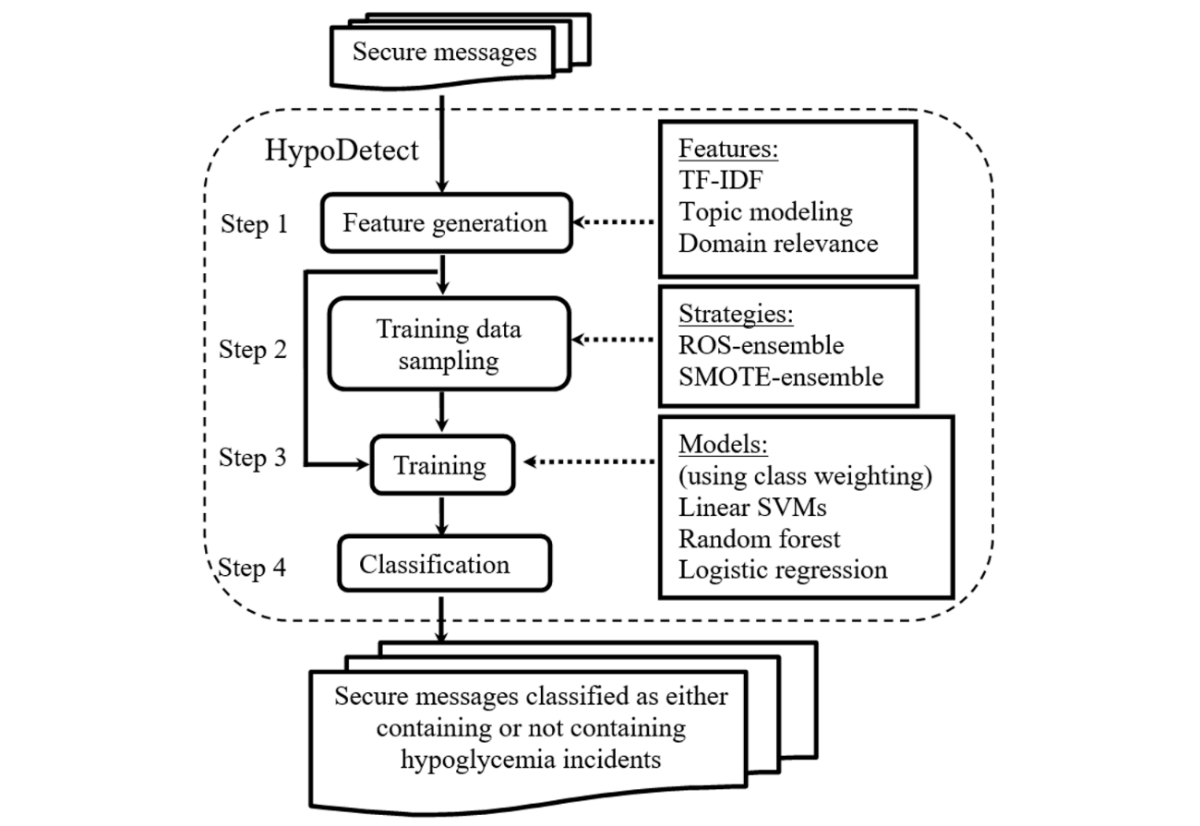
Workflow of HypoDetect. ROS: random oversampling; SMOTE: synthetic minority oversampling technique; SVM: support vector machine; TF-IDF: term frequency-inverse document frequency.

HypoDetect processed the data in 4 steps: feature generation, training data sampling, training, and classification (Figure 1). We investigated 2 oversampling strategies in mitigating data imbalance (Figure 1, step 2) and 3 machine learning methods for text classification (Figure 1, step 3, trained using class weighting).

Cost-sensitive learning and data sampling are 2 strategies that have been widely used to address the problem of data imbalance [28,35,36], including problems in the clinical domain [29-32]. Cost-sensitive learning addresses data imbalance at the algorithm level by associating high costs with misclassifying minority examples (also called class weighting) when training machine learning models. Sampling methods modify the training data to make them balanced and thus suitable for standard learning algorithms. Details are as follows.

#### Data Sampling to Reduce Data Imbalance

##### Random Oversampling

This method randomly samples minority examples (positive examples in our case) with replacement to increase the number of positive examples. Previous work [31] found that sampling the training set to be completely balanced (ie, having equal numbers of positive and negative instances) was an effective strategy. We therefore adopted this strategy for random oversampling and SMOTE (described below) in our study.

##### Synthetic Minority Oversampling Technique

**Figure 2:**
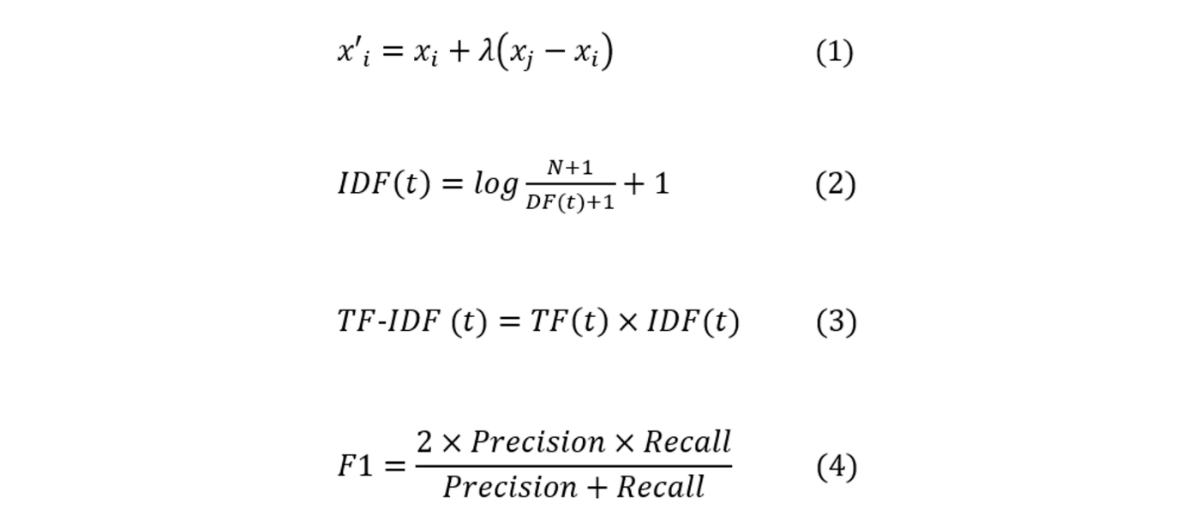
Equations for (1) synthetic minority oversampling technique, (2) inverse document frequency (IDF), (3) term frequency-inverse document frequency (TF-IDF), and (4) F1 measure.

Instead of randomly oversampling minority (positive) examples, SMOTE [37] creates “synthetic” positive examples. Specifically, for each positive example *x*_*i*_, SMOTE generates a new example by using this example and its *k* positive-class nearest neighbors in the feature space, as defined in equation (1) (Figure 2), where *x′*_*i*_ is the new example synthesized from the positive example *x*_*i*_ and the example *x*_*j*_ that is randomly selected from *x*_*i*_’s *k* nearest neighbors, and λ is a random value ranged in [0,1]. We set *k*=5 by following previous work [37].

By its definition in equation (1), SMOTE usually will not remove a word feature (ie, set the feature value to 0) from a synthesized message if the word occurred in the positive message used to generate the synthesized message. Another property of SMOTE is that it can enrich the representation of a message by using additional words that occurred in messages similar to this message. This treatment may alleviate the data sparsity problem that often occurs when using word features.

SMOTE is widely used for learning from imbalanced data due to the simplicity of its sampling procedure and its robustness when applied to different types of problems [38], including clinical classification problems [31,32]. By comparing SMOTE with its 3 variations (borderline SMOTE, support vector machine [SVM] SMOTE, and adaptive synthetic sampling approach) in our preliminary experiments using the first fold of our data, we found that SMOTE worked consistently better with the 3 machine learning algorithms used by HypoDetect. We therefore chose SMOTE for this study.

##### Ensembled Oversampling Methods

We extended each oversampling method to an ensemble version to improve model robustness. Specifically, during the training phase, we ran an oversampling method on the training set 10 times to train 10 models. We then classified the test examples by voting from the 10 models.

#### Machine Learning Models and Class Weighting

While deep learning has shown success in text classification [39], it mostly worked well when the training dataset is large. Due to the knowledge bottleneck challenge, the clinical training dataset is frequently much smaller. Recent work showed that feature-based supervised machine learning approaches outperformed deep learning approaches in certain clinical classification tasks [40]. Therefore, we experimented with 3 feature-based machine learning algorithms (details in Multimedia Appendix 2) that have been widely used and are the state-of-the-art for text classification: linear support vector machines [41], random forest [42], and logistic regression.

We used balanced class weighting when training the cost-sensitive machine learning models. Specifically, we weighted each class by the reciprocal of the number of training instances belonging to that class.

#### Features for Machine Learning

##### Term Frequency-Inverse Document Frequency

TF is the number of occurrences of a word in each individual secure message. IDF and TF-IDF are calculated by equations (2) and (3) (Figure 2), where *t* is a word, *DF(t)* is the number of secure messages in a data collection that contained *t* (also called document frequency of *t*), and *N* is the total number of secure messages in the data collection. We computed IDF on the 3000 secure messages used in this study. We removed words that occurred in the stop-word list from scikit-learn [43] or occurred less than 3 times in our secure message corpus. In total, we generated 5910 normalized, real-valued TF-IDF features.

##### Topic Features

Topic features, as represented by *P* (*topic*_*i*_| *d*) (*i*=1, 2,… *K*), are real-valued features in [0,1] to indicate the probability of the *i* th *topic* given a document *d* (ie, a secure message in our case). *K* is the number of topics used in topic modeling.

We first used the latent Dirichlet allocation algorithm [44] implemented by the Machine Learning for Language Toolkit (MALLET) [45] to train a topic model on 10,000 secure messages that were randomly selected from the same data pool we used to select the evaluation data. We then applied the topic model to the 3000 secure messages to obtain the topic features, that is, the topic distribution over each secure message. We set the topic number *K* to 100 after manually assessing the informativeness and granularity of topics generated by using different *K*s (*K*=20, 30, 50, 100, 200). We set other hyperparameters of topic modeling to default values. For example, we set the concentration parameters Alpha (for distribution of topics per document) and Beta (for distribution of words per topic) to 50/*K* and 0.01. The first table in Multimedia Appendix 3 shows examples of topics generated by topic modeling.

##### Domain-Relevance Features

We defined domain-relevance features by word embeddings and predefined domain-specific keywords.

Word embeddings are distributed vector representations of words. Word embeddings have emerged as a powerful technique for word representation and proved beneficial in a variety of biomedical and clinical NLP tasks. We used word2vec software to create the word embeddings [46,47] and trained word2vec using 4.86 million electronic health record notes, including progress reports, discharge summaries, history and physical reports, and consult notes, from UMass Memorial Medical Center, Worcester, MA, USA. We used 200-dimension vectors by following Jagannatha et al [48] and Pyysalo et al [49]. We used the continuous bags of words model with a window set of 8, negative sampling (25 negative samples for each positive sample), and a subsampling threshold of 0.0001 for training.

To generate the domain-relevance features, we manually created 8 topics by keywords describing hypoglycemia symptoms or incidents (see the second table in Multimedia Appendix 3). We then used these topics to create 28 features in the following steps.

We first included 8 binary-valued features indicating whether the message contained a word belonging to a topic. Then, we included 4 binary features indicating whether the message contained a domain-specific topic word, a number, a number lower than 70, and keywords such as hypoglycemia, low sugar, and low blood sugar.

To generate real-valued features, we represented a topic by the average word embeddings of its topic words [48,50]. For each secure message and each topic, we computed the cosine similarities between this topic and the words in this message and chose the maximum similarity score as the feature value for this topic. This way, we obtained 8 real-valued features associated with 8 topics. We then normalized the 8 features to obtain another 8 real-valued features.

### Baseline Systems

To examine the effectiveness of using cost-sensitive learning and oversampling to mitigate the challenge of data imbalance, we compared the HypoDetect systems that use these strategies with 3 types of baselines: (1) a rule-based classifier, (2) the systems that did not treat data imbalance, and (3) the systems that used ensembled undersampling.

The rule-based method classified a message as positive if it satisfied either of the following criteria: (1) it contained the keyword low blood pressure, low sugar, hypoglycemia, or hypoglycemic; or (2) it contained the keyword sugar or glucose, and contained at least two typical symptoms related to hypoglycemia (details in Multimedia Appendix 4).

Ensembled undersampling extends undersampling that randomly selects a subset of examples from the majority class to balance the training data. It has been shown to be effective or even better than oversampling for some classification tasks [51]. However, this method may not work well when the number of positive instances is small and the feature space is large.

### Experimental Settings

We used 2-layer 10-fold cross-validation to develop and evaluate supervised learning systems. Specifically, we divided the 3000 messages into 10 folds using stratified random sampling. Each fold had 300 messages, with 10 to 12 positive messages. For each fold, we used the remaining 9 folds to train the system and evaluated the system on this fold. By repeating this procedure for each fold, we obtained the evaluation results on the full dataset. When training the system, we used 10-fold cross-validation on the training data to find the optimal hyperparameters. This approach allows for all the data to be used as the evaluation set while ensuring that the training, validation, and evaluation data are separated.

When applying the data sampling techniques, we only sampled the training data by following Blagus and Lusa’s work [32]. After oversampling, the training set for each fold contains 5196 (2598 positive and 2598 negative) or 5194 (2597 positive and 2597 negative) examples. After undersampling, the training set for each fold contains 204, 206, or 208 (with equal numbers of positive and negative examples) examples.

We used the open source software scikit-learn [43], version 0.19.1, to build the systems and develop the ensembled sampling techniques.

We report our evaluation results at the corpus level (ie, first merging the system outputs from the 10 folds and then calculating the evaluation metrics) in the Results section and the fold-level results in Multimedia Appendix 5. In addition, in the third table in Multimedia Appendix 5, we provide statistics (mean, standard deviation, minimum, and maximum) of the performance scores of individual classifiers used by the ensembled oversampling models.

### Evaluation Metrics

#### Sensitivity (Recall), Specificity, Precision, and F1 Score

Sensitivity, or recall, is the number of true positives (ie, secure messages that contained hypoglycemia events and were correctly predicted by the model) divided by the total number of positive instances (ie, total number of secure messages that contained hypoglycemia events).

Specificity is the number of true negatives divided by the total number of negative instances.

Precision is the number of true positives divided by the total number of instances that were predicted to be positive by the model.

The F1 score is the weighted average of precision and recall, as defined by equation (4) (Figure 2). The F1 score takes both false positives (measured by precision) and false negatives (measured by recall) into account. This measure is often used to assess a classifier’s performance on handling uneven class distribution, that is, imbalanced data [28,31,51].

#### Area Under the Receiver Operating Characteristic Curve

This computes the area under the receiver operating characteristic curve (AUC-ROC), which plots the true-positive rate (y-coordinate) against the false-positive rate (x-coordinate) at various threshold settings.

For each ensemble model, we used the mean of the probabilities output by its 10 single models to compute the AUC-ROC. To calculate the AUC-ROC for the rule-based method, we assigned 0 to messages that did not contain hypoglycemia symptoms, 1 to messages that contained hypoglycemia symptoms but did not satisfy the criteria used by the rule-based method to select positive messages (see the Baseline Systems subsection above for the criteria), 2 to messages that satisfied the second criterion used by the rule-based method, and 3 to messages that satisfied the first criterion.

#### Accuracy

Accuracy is the number of correctly classified instances divided by the total number of instances. Although traditionally accuracy is the most common measure for classification, it is less effective and sometimes even improper when measuring performance on imbalanced classes [52,53]. In this paper, we provide accuracy for readers’ interest, but we compared system performance based on other measures (eg, sensitivity and F1 score) [52,53].

### Feature Analysis

We conducted feature ablation experiments to examine the effects of features. Specifically, we first selected the 3 best variants of HypoDetect that used different machine learning algorithms and different strategies to address data imbalance. We then compared these systems with their counterparts that dropped each single type of feature respectively.

To gain some understanding of the effects of individual features, we used a hybrid method to identify indicative features. Our method was motivated by the fact that our system, like typical NLP systems, uses a large number of features that are potentially redundant and may have dependencies among each other. As a result, the feature weights from the full model that used all the features may not accurately reflect a feature’s impact. To address this problem, our method took into account the feature’s adjusted (when used in the full model) and unadjusted (when used alone) effects. Specifically, we first used the best full model to identify 100 features with the largest positive feature weights. We then evaluated 100 single-feature models (which used the same machine learning method as the full model and used only 1 feature) through cross-validation and ranked the 100 features based on the corresponding F1 scores.

### Error Analysis

To identify sources of errors, we analyzed hundreds of false-positive and false-negative instances that were predicted with high confidence by the 3 best variants of HypoDetect.

## Results

### Secure Messages and Patient-Reported Hypoglycemia Events

The interannotator agreement between the 2 annotators on the 100 secure message threads was Cohen kappa=.976.

Our dataset contained 3000 secure messages. The distribution of the number of words contained in these messages was right skewed (the first figure in Multimedia Appendix 5), with a median length of 92 (interquartile range 49-168) words. A total of 2850 (95.00%) of the 3000 messages had fewer than 435 words, and 114 (3.80%) messages were annotated as positive, indicating that the data were highly imbalanced.

Diabetic patients reported both mild and severe hypoglycemia incidents through secure messaging (see Textbox 1 and the first table in Multimedia Appendix 1). As Textbox 1 shows, patients wrote messages in diversified, informal ways (eg, “eating low carb” in example 1 and “blood sugar #” in example 2) and with typos (eg, “Gllipizide,” “stablize,” and “to much” in example 2). In addition, patients often elaborated on symptoms rather than directly reporting blood glucose levels.

### Performance of Different HypoDetect Systems on the Evaluation Set

Corpus-level evaluation (Table 1) showed that logistic regression with class weighting achieved the best AUC-ROC (0.954) and F1 score (0.590). This classifier had a high specificity (0.974) and balanced sensitivity (0.693) and precision (0.513).

The 3 baseline machine learning systems (without treating data imbalance) consistently had very high specificity and very low sensitivity because they classified most examples as negative. Class weighting and oversampling (ROS-ensemble and SMOTE-ensemble) improved the baselines’ sensitivity substantially (0.123-0.728 absolute gains) and their overall performance (as measured by the F1 score and AUC-ROC). Class weighting worked best for linear SVMs and logistic regression, whereas SMOTE-ensemble worked best for random forest.

Undersampling (RUS-ensemble in Table 1) boosted the baselines’ sensitivity even higher but dropped their specificity and precision substantially. The rule-based method had higher sensitivity than the baseline machine learning systems but had lower performance than systems using class weighting or oversampling for all the metrics.

The fold-level evaluation showed similar results (see the first and second tables and the second figure in Multimedia Appendix 5). The individual classifiers used by an ensembled oversampling model had similar performance (the third table in Multimedia Appendix 5).

### Effects of Features

We tested the effects of features on the 3 best variants of HypoDetect, namely linear SVMs with class weighting, random forest with SMOTE-ensemble, and logistic regression with class weighting. The results (Table 2) showed that dropping TF-IDF or domain-relevance features decreased the comprehensive metrics (AUC-ROC, F1 score, and accuracy) of all 3 systems and also decreased most single metrics (especially precision and specificity). Dropping topic features had mixed results. It decreased most metrics for logistic regression with class weighting and random forest with SMOTE-ensemble but increased most metrics for linear SVMs with class weighting.

**Excerpts from 2 secure messages reporting incidents of hypoglycemia.**

Example 1: “Can you tell me what glucose level is too low? The last couple of nights, I’ve woken up in the middle of the night sweating profusely and shaky. I got up to check my blood sugar and it’s been 63 both nights. Is that too low? After testing, I eat a snack and test again, and it goes up, to 73 Wednesday night/Thursday morning and to 70 Thursday night/Friday morning. I’m dieting, I’ve lost 7 pounds since the first of the month, but I’m not really eating low carb. This evening before I go to bed, I’m going to test my blood and if it’s low, eat something before going to bed.”

Example 2: “I took Gllipizide in the am before breakfast and one before dinner. Last night my blood sugars took a dive. I went to sleep and around 11 I woke up sweating and clammy. I took my blood sugar # and it had dropped to 57. My wife quickly brought me sugar tablets and I was able to stablize them at 80. I think this is to much medication.”

**Table 1:** Performance of 3 variants of HypoDetect systems on the evaluation set.

Systems		AUC-ROC^a^	Precision	Sensitivity (recall)	Specificity	F1 score	Accuracy
Rule-based method		0.815	0.284	0.491	0.951	0.360	0.934
**Linear support vector machines**							
	Baseline	0.945	0.614	0.377	0.991	0.467	0.966
	Class weighting	0.952	0.529	0.561	0.980	0.545	0.964
	RUS-ensemble^b^	0.949	0.198	0.921	0.852	0.326	0.855
	ROS-ensemble^c^	0.950	0.559	0.500	0.984	0.528	0.966
	SMOTE-ensemble^d^	0.951	0.564	0.500	0.985	0.530	0.966
**Random forest**							
	Baseline	0.942	0.000	0.000	1.000	0.000	0.962
	Class weighting	0.927	0.428	0.570	0.970	0.489	0.955
	RUS-ensemble	0.928	0.143	0.904	0.787	0.248	0.791
	ROS-ensemble	0.931	0.318	0.728	0.938	0.443	0.930
	SMOTE-ensemble	0.942	0.486	0.596	0.975	0.535	0.961
**Logistic regression**							
	Baseline	0.947	0.660	0.307	0.994	0.419	0.968
	Class weighting	0.954	0.513	0.693	0.974	0.590	0.963
	RUS-ensemble	0.946	0.192	0.912	0.849	0.318	0.851
	ROS-ensemble	0.951	0.536	0.526	0.982	0.531	0.965
	SMOTE-ensemble	0.951	0.566	0.552	0.983	0.559	0.943

^a^AUC-ROC: area under the receiver operating characteristic curve.

^b^RUS-ensemble: ensemble models using random undersampling.

^c^ROS-ensemble: ensemble models using random oversampling.

^d^SMOTE-ensemble: ensemble models using synthetic minority oversampling technique.

**Table 2:** Performance of different HypoDetect systems implemented by using all types of features or by respectively dropping each individual type of feature.

Systems		AUC-ROC^a^	Precision	Sensitivity (recall)	Specificity	F1 score	Accuracy
**Linear support vector machines with class weighting**							
	All	0.952	0.529	0.561	0.980	0.545	0.964
	Without TF-IDF^b^	0.920	0.263	0.737	0.919	0.388	0.912
	Without topic	0.949	0.569	0.579	0.983	0.574	0.967
	Without domain relevance	0.928	0.348	0.623	0.954	0.447	0.941
**Random forest with SMOTE-ensemble^c^**							
	All	0.942	0.486	0.596	0.975	0.535	0.961
	Without TF-IDF	0.938	0.364	0.632	0.956	0.462	0.944
	Without topic	0.935	0.392	0.640	0.961	0.487	0.949
	Without domain relevance	0.901	0.365	0.237	0.984	0.287	0.955
**Logistic regression with class weighting**							
	All	0.954	0.513	0.693	0.974	0.590	0.963
	Without TF-IDF	0.917	0.248	0.754	0.910	0.373	0.904
	Without topic	0.950	0.500	0.640	0.975	0.561	0.962
	Without domain relevance	0.901	0.437	0.579	0.971	0.498	0.956

^a^AUC-ROC: area under the receiver operating characteristic curve.

^b^TF-IDF: term frequency-inverse document frequency.

^c^SMOTE-ensemble: ensemble models using synthetic minority oversampling technique.

We used the best model—that is, logistic regression with class weighting—to analyze fine-grained feature effects. The results from this analysis showed that “low,” “sweating,” “shaking,” “sugar,” and “took” were among the top-10 features. Other top-10 features included 2 topic features (corresponding to topics 37 and 49 in the first table in Multimedia Appendix 3) and 3 domain-relevance features (corresponding to domain-specific topics 3, 4, and 5 in the second table in Multimedia Appendix 3).

## Discussion

### Principal Findings

We developed HypoDetect, an NLP system that automatically detects patient-reported hypoglycemia incidents from secure messages to facilitate early response from health care providers. Despite the challenges caused by imbalanced data and informal language use by patients, HypoDetect using logistic regression with class weighting achieved an AUC-ROC of 0.954 and F1 score of 0.590 on the evaluation set. This system had a high specificity (0.974) and a moderate sensitivity (0.693).

The F1 score is often used to assess the system’s capability to tolerate data imbalance because it is sensitive to data imbalance. This score is usually much lower on imbalanced datasets than on balanced ones [28,54]. F1 scores reported by previous studies on highly imbalanced datasets typically ranged between 0.3 and 0.5 [31,51]. Therefore, the F1 score of 0.590 achieved by our system is very promising.

Our work has clinical relevance. As introduced previously, secure messaging is intended for exchanging nonurgent information. Secure messaging also follows a triage process. The messages are viewed first by a nurse. If he or she determines it is necessary, the message will be forwarded to the clinician for review. This process can lead to both underreporting of and delayed responses to hypoglycemia events. First, the nurse may address a secure message about hypoglycemia and then close out the message, so that the clinician is not aware of the incident and the incident is not recorded in the patient’s record. Second, the triage process means that responses from clinical teams could be delayed, putting patients at higher risk for severe consequences. Our system has the potential to serve as a surveillance tool to support a proactive and timely response in such situations and, therefore, improve patient safety.

Previous work predicted the occurrence of hypoglycemia in a future period by learning from physiological data, such as monitored glucose levels and heart rate variability [55-60]. In contrast, our goal was to identify hypoglycemia events that have already happened and have been reported by patients. The inputs for our system were patients’ descriptions about the adverse events, which were mainly symptoms and often did not contain information about blood sugar levels.

Previous work on automatic classification of secure messages focused on information needs and did not address data imbalance [25-27]. Our work contributed to this literature by introducing a new task and by investigating strategies for treating data imbalance. Paragraph embeddings were shown useful for classifying information needs in secure messages [27]. In the future, we will study the effects of using document embeddings as learning features for our task.

There has been active research in using NLP to detect or facilitate manual review of adverse drug events in unstructured electronic health record notes [40,61-64]. The prior work identified adverse events at the entity (eg, medical terms representing side effects of a drug) or relation (eg, a pair of terms that represent a drug and its side effects) level. In this study, we annotated hypoglycemia events at the message-thread level because patient-reported hypoglycemia events often lacked pivot terms and were composed of a set of symptoms and pertinent context. Sentence-level annotation may further improve system performance, which we will explore in the future.

### Effects of Treating Data Imbalance

Our results showed that cost-sensitive learning (ie, class weighting) and SMOTE-ensemble were most effective in boosting system performance on imbalanced data (Table 1). Without treating data imbalance, the baseline systems failed to detect most positive examples. Class weighting and oversampling improved the sensitivity of all 3 variants of HypoDetect substantially. As a tradeoff, the specificity and precision decreased to a certain extent, but the overall effects (as measured by F1 score and AUC-ROC) were positive. Because oversampling increases the size of training data, it is computationally more expensive than class weighting.

Oversampling (ROS-ensemble and SMOTE-ensemble in Table 1) performed much better than undersampling (RUS-ensemble in Table 1) on our task when measured by the AUC-ROC and F1 score that consider both false positives and false negatives. Like typical text classifiers that use word features, our system uses thousands of features. However, the training set created by undersampling contained only about 200 examples, which was likely too few to train the system.

Patient-reported hypoglycemia needs to be evaluated quickly to avoid severe consequences. Therefore, systems with low sensitivity (eg, the baseline systems in our study) cannot be used for surveillance. On the other hand, systems with high sensitivity but low precision (eg, the systems using ensembled undersampling) would generate many false alarms, adding undue burden on already time-strapped health care providers. An ideal system for hypoglycemia detection would have high sensitivity and precision. To achieve this goal, we will explore ensemble methods that combine different types of systems (eg, systems with high sensitivity and systems with high precision) in our future work.

### Effects of Features

Our results showed that TF-IDF, topic features, and domain relevance all contributed to system performance.

TF-IDF has been widely used for text classification. However, one disadvantage of TF-IDF is that it ignores semantic information and treats words with the same or similar meanings as separate entities. As a result, there are often thousands of TF-IDF features, posing challenges for machine learning when the training set is of small or moderate size.

Topic features cluster terms into a small set of semantically related groups, which helps alleviate the data sparseness problem to a certain extent. Topic features and their variants have proved useful for text classification, including categorizing clinical reports [65-67]. However, automatically induced topics may not be accurate and may lose fine-grained information for document classification. Therefore, a combination of both types of features is likely more robust.

The domain-relevance features are new features that we designed for this task. Our results suggested that knowledge-driven features can effectively improve system performance for domain-specific classification tasks.

### Error Analysis

First, the systems often failed on cases that required discourse-level comprehension or human knowledge. For example, they tended to classify positive messages as negative if the messages contained irrelevant information. They also often classified negative messages as positive if the messages mentioned “blood sugar” and symptoms that looked similar to but were not caused by hypoglycemia (eg, example 4 in the second table in Multimedia Appendix 1). We expect that more annotated data will help reduce this type of error.

Second, our systems did not have specific treatments on negation and questions and therefore could be confused by messages that mentioned symptomatic terms in a negative mode or mentioned “low sugar level” or “hypoglycemia” in hypothetical questions (eg, examples 2 and 3 in the second table in Multimedia Appendix 1). Negation and question detection systems could be integrated to reduce this type of error.

Third, the systems often failed to extract glucose testing results when patients reported these numbers in informal ways. Example 4 in the first table in Multimedia Appendix 1 is a typical example, where the patient mentioned “This morning I had a 66.” One way to reduce this type of error is to develop another classifier to judge whether the blood sugar level is normal or abnormal and then use the classification results as a feature for our hypoglycemia classifier.

### Limitations

Within the scope of this study, we annotated hypoglycemia incidents based on information solely in secure messages and treated instances that lacked clear context as negative. Clinic visit notes from around the time of the message may provide more information to reduce uncertainty, which we will study in the future. In this study, we used keywords to sample more positive examples because the positive examples retrieved by random sampling were too few to train and evaluate supervised systems. This strategy may affect the system’s performance in a real-world setting. We included the original, randomly sampled 1000 messages (with 1 positive example only) into our training data as a way to alleviate this problem.

### Conclusions and Future Work

We developed HypoDetect, an NLP system to automatically identify patient-reported hypoglycemia incidents from secure messages to facilitate early response and corrective actions by clinical teams. Despite the challenge of data imbalance, HypoDetect using class weighting or SMOTE-ensemble achieved promising results on this task. In future, we will investigate advanced data-driven methods, including active learning and document embeddings, to improve HypoDetect.

## Appendix

Multimedia Appendix 1 Annotation guideline and examples of positive and negative instances for hypoglycemia incidents in our dataset.

Multimedia Appendix 2 Machine learning algorithms used by the HypoDetect system.

Multimedia Appendix 3 Supplemental information about features used in this study.

Multimedia Appendix 4 Keywords used by the rule-based method.

Multimedia Appendix 5 Supplemental results from this study.

## Abbreviations

AUC-ROC: area under the receiver operating characteristic curve
HypoDetect: Hypoglycemia Detector
ICD-9: International Classification of Diseases, Ninth Revision
ICD-10: International Classification of Diseases, Tenth Revision
MALLET: Machine Learning for Language Toolkit
NLP: natural language processing
SMOTE: synthetic minority oversampling technique
SVM: support vector machine
TF-IDF: term frequency-inverse document frequency
VA: Veterans Affairs
