# Supplementary material for: Detecting Hypoglycemia Incidents Reported in Patients’ Secure Messages: Using Cost-Sensitive Learning and Oversampling to Reduce Data Imbalance
Source: J Med Internet Res. 2019 Mar 11;21(3):e11990. doi: 10.2196/11990 (PMC6431826; doi:10.2196/11990)
Supplement: Multimedia Appendix 1 [file jmir_v21i3e11990_app1.pdf]

Multimedia Appendix 1: Annotation guideline and examples of positive and negative instances for hypoglycemia incidents in our dataset

Guidelines for Annotating Secure Messages Where Patients Reported Hypoglycemia Incidents

1. Task: to identify secure message threads in which patients reported hypoglycemia incidents

2. Operational rules for annotation:

(1) A patient's secure message is positive if either of the following conditions holds:

a. the message mentions a blood glucose level <70mg/dl

b. the message describes typical hypoglycemia symptoms that cannot be contextually attributed to other possible causes (e.g., high blood sugar, low blood pressure, and other unrelated illness)

(2) A secure message thread is positive if it contains a positive patient secure message.

Note:
Blood glucose level < 70mg/dl is not a hard requirement for identifying positive messages for the following reasons:

a. There is a variability in the precision of patient-reported blood glucose level (e.g., the quality and type of the blood sugar monitor and the patient's skill in measuring blood glucose can affect the measurement results)
b. A patient may report only hypoglycemic symptoms but not blood glucose levels

Annotation Examples

Note: For each message, we only showed the content most relevant to the annotation judgement.

Table A1-1. Positive examples for hypoglycemia incidents in the dataset.

No.	Examples
1	I slipped into unconsciousness and he was unable to wake me after several attempts. …The two paramedics worked on me for over 45 minutes… upon their arrival my blood sugar level was… 40.
2	I don't like messageing all the time, but I have serious questios about my meds. Dr. xxx

	prescribed Glipizide, 10mg tabs to be taken along with Metformin. I believe this was to reduce my A1C number. After discussing this with you last week, I took the first pill along with the Metformin with my evening meds. The next morning I didn't feel well, dizzy, weak, shakey. I didn't think too much about it then. I took my morning meds, including the Glipizide and Metformin. Late morning, early afternoon, I was really feeling weak, dizzy and really shakey. I checked my blood sugar and it was 71mg/dL. Normal readings are in the 110 to 120 range.
3	Are you sure about the doubling of my glipizide...? With only 5mg, if I forget to eat right away, I start getting the shakes, etc. As discomforting as it is when that happens, what will happen if I forget to eat right away when I'm taking 10mg's...? I'm afraid I might pass out, because it comes on really quick...?
4	In light of my good A1C on **date**, on **date**, xxx and I discussed lowering my insulin dose from 20u back down to 18u. She said stay at 20u unless my home test readings warranted the change. This morning I had a 66, which I think is the lowest I've ever had. Oddly, I did not feel shaky as I usually do when it's down around 90. So, unless you object, I will revert to 18u immediately.

Table A1-2. Negative examples for hypoglycemia incidents in the dataset.

No.	Examples
1	I have completely stopped the bupropion. I have cut the Metforeman to 500 mg a day. I am scheduled to see Dr. xxx on **date**. My headaches have stopped. My sugar is under control at 89-109.
2	After talking with you yesterday concerning my B.S. :) Blood sugar. I have came down with the following symptoms. Stinging at the beginning of urinating, urinating every 3-4 hours, on and of sweats, dry mouth, lower abdominal discomfort (0 pain), difficulty going and staying asleep and boy am I hungry … No fever, No dizzy and no shaking. Hypoglycemic?
3	I do have a question? a couple of nights ago before bed my sugar was 228 and when i got up the next morning it went down 100 point to 127. Tonight my sugar is 168 will it go down by 100 each night and will 68 be kind of low? What should my sugar be at at bed time or whats the lowest it should be before bed?? Just scared of it going down too low while i'm asleep What are the possibilities?? Should have asked this question at the class last month.
4	I think the problem is caused by Rifaximin. I just started it and was taking one 550 mg at night but I am supposed to take that twice a day and started the morning dose today. I feel a little weak and shaky but I think i am doing better than earlier. This is the worst episode I have ever had with blood sugar. I am also exhausted from taking care of xxx. My legs are killing me. I am weak and slightly short of breathe but better than earlier. If absolutely necessary I will call EMT but I will not go to ER.
