# Supplementary material for: Detecting Hypoglycemia Incidents Reported in Patients’ Secure Messages: Using Cost-Sensitive Learning and Oversampling to Reduce Data Imbalance
Source: J Med Internet Res. 2019 Mar 11;21(3):e11990. doi: 10.2196/11990 (PMC6431826; doi:10.2196/11990)
Supplement: Multimedia Appendix 2 [file jmir_v21i3e11990_app2.pdf]

Multimedia Appendix 2: Machine learning algorithms used by the HypoDetect system

Logistic Regression and Linear Support Vector Vectors

Logistic regression (LR) and linear support vector machines (SVMs) are both linear classifiers but are trained based on different principles.

LR aims to maximize the likelihood L of generating the training data given model parameters, as defined by equation (1).

$$P(y = 1|x) = f(w^T x + b)$$

$$L = \sum_{i=1 \text{ to } N} P(y_i|x_i)^{y_i}(1 - P(y_i|x_i))^{1-y_i} - \lambda\|w\|_2^2 \qquad (1)$$

where w and b are parameters for linear transformation, N is the total number of training examples and λ is the regularization parameter to avoid overfitting. During training, we searched λ in [0.01, 0.1, 1, 10, 100] to maximize the F1 score by cross validation on the training set.

Linear SVMs aim to find the separating hyperplane that maximizes the distance (margin) between the closest points (the support vectors) from the two classes. If a data point is not a support vector, it does not affect model parameters of SVMs.

The objective function of linear SVM is defined by equation (2)

$$\min_{w,b,\varepsilon_i} w^T w + C\left(\sum_{i=1 \text{ to } N} \varepsilon_i + \|w\|_2^2\right)$$
$$\text{s.t.}: y_i(w^T x_i + b) \geq 1 - \varepsilon_i\,,\ \varepsilon_i \geq 0 \qquad (2)$$

where w and b are parameters for linear transformation, N is the total number of training examples, and C is the regularization parameter for the error term and the feature weights. We searched C in [0.01, 0.1, 1, 10, 100] to maximize the F1 score by cross validation on the training set.

Random Forest

Random Forest (RF) [1] combines multiple decision trees for classification. It extends the idea of "bagging" [2] with a random selection of features [3–5] to improve robustness and generalizability.

In this study, assuming s is a secure message, the prediction of RF on s, $\hat{f}(s)$, is calculated by (3),

$$\hat{f}(s) = \frac{1}{T}\sum_{i=1}^{T}\hat{f}_i(s) \quad (3)$$

where $\hat{f}_i(s)$ is the possibility of s containing a hypoglycemia event as predicted by the ith tree among T decision trees built for RF.

Each single tree $\hat{f}_i$ is built on N examples randomly sampled with replacement from the training set with sample size N. For a new example s, RF assigns s to a leaf node of each individual decision tree by applying the decision rules learned from the training phase. $\hat{f}_i(s)$ is calculated as the fraction of positive training examples in the leaf node of the ith decision tree where s is assigned. The class label of s predicted by RF is a vote by the trees in the forest, weighted by their probability estimates $\hat{f}_i(s)$. In our case, if $\hat{f}(s)$ calculated by (3) is greater than 0.5, then s is classified as a positive example (i.e., containing a hypoglycemia event).

We used scikit-learn [6] to develop RF. We set three parameters, the number of decision trees in the forest (i.e., T in Eq. (3)), the class weighting method (which is used to balance the number of positive and negative examples when building a decision tree) and the maximum depth of the trees, by cross-validation on the training set and used the default values of other parameters.

References:

1. Breiman L. Random forests. Mach Learn 2001;45(1):5–32.

2. Breiman L. Bagging predictors. Mach Learn 1996;24(2):123–140.

3. Ho TK. Random decision forests. Doc Anal Recognit 1995 Proc Third Int Conf On IEEE; 1995. p. 278–282.

4. Amit Y, Geman D. Shape quantization and recognition with randomized trees. Neural Comput 1997;9(7):1545–1588.

5. Ho TK. The random subspace method for constructing decision forests. IEEE Trans Pattern Anal Mach Intell 1998;20(8):832–844.

6. Pedregosa F, Varoquaux G, Gramfort A, Michel V, Thirion B, Grisel O, Blondel M, Prettenhofer P, Weiss R, Dubourg V, others. Scikit-learn: Machine learning in Python. J Mach Learn Res 2011;12(Oct):2825–2830.
