# Supplementary material for: Detecting Hypoglycemia Incidents Reported in Patients’ Secure Messages: Using Cost-Sensitive Learning and Oversampling to Reduce Data Imbalance
Source: J Med Internet Res. 2019 Mar 11;21(3):e11990. doi: 10.2196/11990 (PMC6431826; doi:10.2196/11990)
Supplement: Multimedia Appendix 3 [file jmir_v21i3e11990_app3.pdf]

Multimedia Appendix 3: Supplemental information about features used in this study

Table A3-1. Example topics generated by topic modeling.

Topic id	Topics	Keywords
10	Diet/food	cheese cup chicken salad lunch toast breakfast coffee oz sandwich oatmeal calories butter dinner milk protein fiber egg eggs rice bread carbs beef raisins fruit peanut total large turkey potato
12	Other disease and symptoms	infection ear throat antibiotic coughing antibiotics nose sinus days nasal ER spray clear cold allergy prescribed sore bad prednisone temp ears urinary infections dry sinuses congestion worse wheezing yellow finished
15	Other disease and symptoms	kidney disease renal liver joint dialysis acute damage evidence transplant degenerative treat stones stone narrowing shows joints potential post chondrocalcinosis inflammation findings kidneys present include moderate affected gene NSAIDs fracture
24	Appointment and referral	consult Dr. clinic appt appointment referral request therapy general put pt schedule make urology call evaluation issues place appts podiatry park heard dermatology requested told ortho order choice neuro pulmonary
37	Blood sugar measurement	sugar readings day morning reading low high eating insulin taking night evening eat hours glucose fasting general levels breakfast meal units average dinner metformin range pm lunch meals numbers running
40	Inquiry	message secure send messaging call questions Mr. clinical contact pharmacy free clinic feel email specialist phone reply pharmacist pharmd concerns Ms. messages center information sincerely telephone forward response office number

49	Warning symptoms (e.g., dizzy, weak, tired)	feel feeling night sugar felt morning dizzy low bed started inquiry day bad water yesterday checked tired pm woke resting weak wake eat hours BP
54	Cancer	cancer prostate risk bd bb biopsy bl bladder increased radiation urology linked based evaluation lung chemo removed bleeding cardiovascular benign treatments tissue possibly breast psa enlarged inflammation urologist bt oncology
67	Diabetes medications	metformin mg glipizide sugar taking daily day diabetes Dr inquiry increase start Victoza sugars morning visit results fasting continue dosage insulin started Saxagliptin tablets glyburide control increased pill advise high
72	Activity	walk walking time minutes inquiry times bed sitting long exercise home standing fall shower sit miles move chair due lost hours fine unable car finally stand driving routine rest scale

Table A3-2. Keywords used to generate domain-relevance features.

Topic No.	Keywords
1	blur, confused, dizzy, headache, hungry, pale, shake, shaking, sleepy, sweat, weak
2	dose, doses
3	drop, down, low, decrease, below, lower, lowest, decreased
4	glucose, sugar
5	hypoglycemia, hypoglycemic
6	increase, above, over, highest, high, higher, increased
7	episode, episodes
8	incident, incidents
