# Supplementary material for: Detecting Hypoglycemia Incidents Reported in Patients’ Secure Messages: Using Cost-Sensitive Learning and Oversampling to Reduce Data Imbalance
Source: J Med Internet Res. 2019 Mar 11;21(3):e11990. doi: 10.2196/11990 (PMC6431826; doi:10.2196/11990)
Supplement: Multimedia Appendix 4 [file jmir_v21i3e11990_app4.pdf]

Multimedia Appendix 4: Keywords used by the rule-based method

Symptom Category No.	Keywords
1	blur
2	confused, confusion
3	dizzy, dizziness, light headedness, light headed, light-headed, light-headedness
4	shaking, shake
5	sweat
6	weak, weakness
7	hunger, hungry
8	nervousness, anxiety
9	sleepiness
10	difficulty speaking
11	loss of consciousness
