# Supplementary material for: Detecting Hypoglycemia Incidents Reported in Patients’ Secure Messages: Using Cost-Sensitive Learning and Oversampling to Reduce Data Imbalance
Source: J Med Internet Res. 2019 Mar 11;21(3):e11990. doi: 10.2196/11990 (PMC6431826; doi:10.2196/11990)
Supplement: Multimedia Appendix 5 [file jmir_v21i3e11990_app5.pdf]

Multimedia Appendix 5: Supplemental results from this study

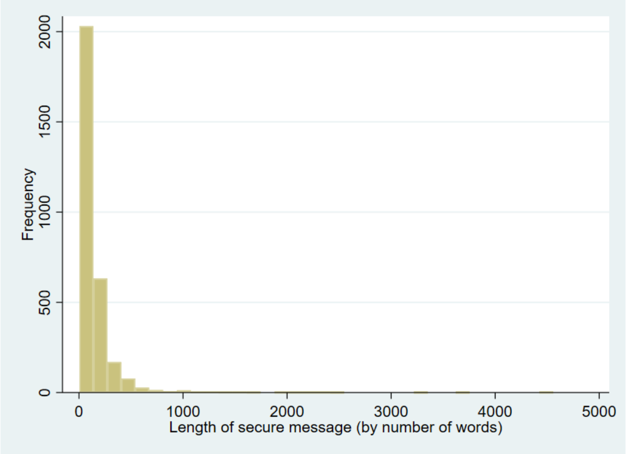

Figure A5-1. Distribution of the length of secure messages. 3000 messages in total, with a median length of 92 (interquartile range=(49, 168)). 2850 (95%) of the 3000 messages had fewer than 435 words.

Table A5-1. Performance of three variants of HypoDetect systems on the evaluation set, averaged by folds in 10-fold cross-validation.

Systems		AUC_ ROC	Precision	Sensitivity (Recall)	Specificity	F1	Accuracy
**Rule-based**		0.815 (0.068)	0.288 (0.090)	0.493 (0.160)	0.951 (0.010)	0.363 (0.113)	0.934 (0.014)
**Linear SVMs**							
	**Baseline**	0.944 (0.038)	0.629 (0.218)	0.378 (0.137)	0.991 (0.006)	0.462 (0.151)	0.967 (0.008)
	**Class weighting**	0.951 (0.034)	0.551 (0.154)	0.566 (0.154)	0.980 (0.010)	0.544 (0.118)	0.964 (0.010)
	**RUS-ensemble**[a]	0.949 (0.039)	0.199 (0.028)	0.921 (0.110)	0.852 (0.013)	0.326 (0.043)	0.855 (0.015)
	**ROS-ensemble**[b]	0.949 (0.035)	0.570 (0.160)	0.503 (0.156)	0.984 (0.007)	0.523 (0.135)	0.966 (0.009)
	**SMOTE-ensemble**[c]	0.950 (0.035)	0.573 (0.158)	0.503 (0.156)	0.985 (0.007)	0.525 (0.135)	0.966 (0.008)
**Random Forest**							

	**Baseline**	0.943 (0.031)	0.000 (0)	0.000 (0)	1.000 (0)	0.000 (0)	0.962 (0.002)
	**Class weighting**	0.927 (0.058)	0.435 (0.133)	0.574 (0.180)	0.970 (0.010)	0.490 (0.140)	0.955 (0.013)
	**RUS-ensemble**	0.929 (0.046)	0.145 (0.022)	0.905 (0.109)	0.787 (0.024)	0.249 (0.036)	0.791 (0.024)
	**ROS-ensemble**	0.932 (0.046)	0.321 (0.080)	0.733 (0.188)	0.938 (0.012)	0.444 (0.105)	0.930 (0.015)
	**SMOTE-ensemble**	0.942 (0.042)	0.490 (0.113)	0.600 (0.144)	0.975 (0.007)	0.535 (0.120)	0.961 (0.010)
**Logistic Regression**							
	**Baseline**	0.952 (0.037)	0.673 (0.267)	0.310 (0.138)	0.994 (0.005)	0.415 (0.167)	0.968 (0.009)
	**Class weighting**	0.953 (0.036)	0.540 (0.151)	0.696 (0.156)	0.974 (0.012)	0.593 (0.117)	0.963 (0.013)
	**RUS-ensemble**	0.947 (0.041)	0.193 (0.029)	0.913 (0.113)	0.849 (0.014)	0.319 (0.044)	0.851 (0.016)
	**ROS-ensemble**	0.950 (0.036)	0.553 (0.171)	0.528 (0.162)	0.982 (0.009)	0.528 (0.139)	0.965 (0.010)
	**SMOTE-ensemble**	0.951 (0.036)	0.586 (0.146)	0.563 (0.145)	0.983 (0.008)	0.561 (0.112)	0.967 (0.008)

[a]RUS-ensemble: ensemble models using random under-sampling
[b]ROS-ensemble: ensemble models using random over-sampling
[c]SMOTE-ensemble: ensemble models using Synthetic Minority Over-sampling Technique

Table A5-2. Performance comparison between the best HypoDetect systems and other systems, as measured by F1 score.[a]

System A	System B		
	Linear SVMs: class weighting	Random Forest: SMOTE-ensemble	Logistic Regression: class weighting
Rule based	*P<.001*	*P<.001*	*P<.001*
Linear SVMs			
Baseline	*P=.004*		
RUS-ensemble[b]	*P<.001*		
ROS-ensemble[c]	*P=.09*		
SMOTE-ensemble[d]	*P=.1*		
Random Forest			
Baseline		*P<.001*	
RUS-ensemble		*P<.001*	

ROS-ensemble		*P*<.001	
Class weighting		*P*=.06	
Logistic Regression			
Baseline			*P*=.002
RUS-ensemble			*P*<.001
ROS-ensemble			*P*=.02
SMOTE-ensemble			*P*=.08

[a]one-sided paired t-test. Treating F1 score on each fold as an observation and paired the F1 scores of two systems for the same fold. H_0: F1 score of system A == F1 score of system B. H_a: F1 score of system A < F1 score of system B
[b]RUS-ensemble: ensemble models using random under-sampling
[c]ROS-ensemble: ensemble models using random over-sampling
[d]SMOTE-ensemble: ensemble models using Synthetic Minority Over-sampling Technique

(a)

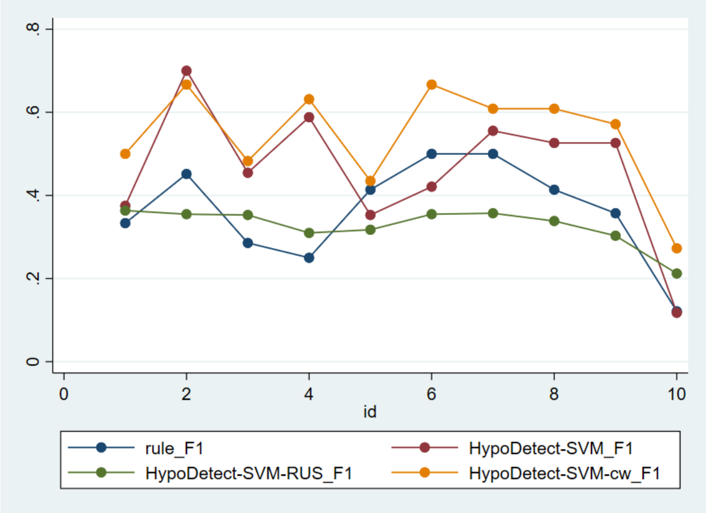

(b)

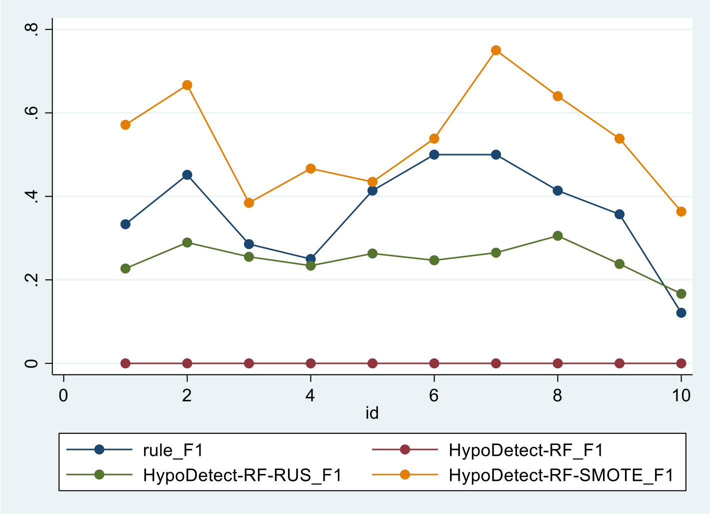

(c)

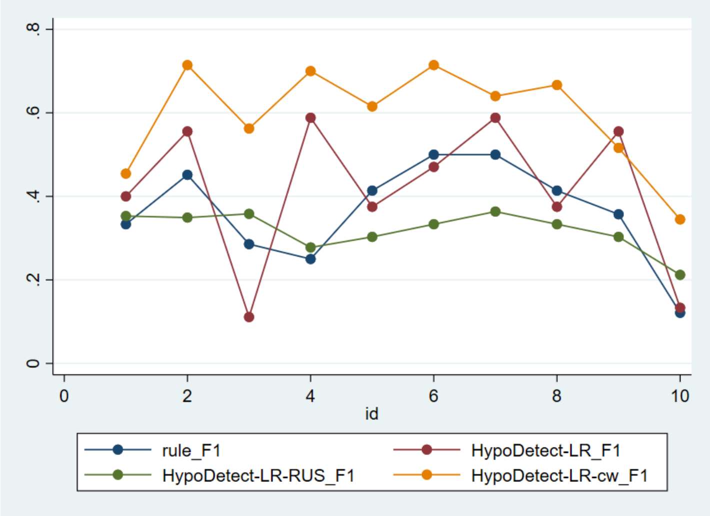

Figure A5-2. Comparing F1 scores of the best HypoDetect system and the baseline systems on each fold. (a): Linear SVMs, (b): Random Forest, and (c): Logistic Regression. Blue (baseline): rule-based method; Red (baseline): machine learning models without treating data imbalance; Green (baseline): ensemble models using random under-sampling (RUS); Orange (best): models using class weighting (cw) or ensemble models using Synthetic Minority Over-sampling Technique (SMOTE).

Table A5-3. Performance of individual classifiers used in ensembled over-sampling HypoDetect systems.

**Performance Metrics**		**Linear SVM**		**Random Forest**		**Logistic Regression**	
		**ROS-ensemble**[a]	**SMOTE-ensemble**[b]	**ROS-ensemble**	**SMOTE-ensemble**	**ROS-ensemble**	**SMOTE-ensemble**
**AUC-ROC**							
	**Mean (SD)**	0.951 (0.000)	0.951 (0.000)	0.930 (0.002)	0.941 (0.002)	0.951 (0.000)	0.951 (0.000)
	**Minimum**	0.950	0.950	0.927	0.938	0.951	0.951
	**Maximum**	0.951	0.951	0.934	0.944	0.951	0.952
**Precision**							
	**Mean (SD)**	0.560 (0.004)	0.567 (0.004)	0.302 (0.007)	0.470 (0.016)	0.541 (0.003)	0.565 (0.006)
	**Minimum**	0.554	0.560	0.290	0.450	0.531	0.557
	**Maximum**	0.564	0.574	0.313	0.500	0.544	0.577
**Sensitivity (Recall)**							

	**Mean (SD)**	0.502 (0.007)	0.504 (0.007)	0.711 (0.008)	0.601 (0.017)	0.534 (0.006)	0.561 (0.000)
	**Minimum**	0.491	0.491	0.693	0.588	0.526	0.561
	**Maximum**	0.509	0.518	0.719	0.640	0.544	0.561
**Specificity**							
	**Mean (SD)**	0.984 (0.000)	0.985 (0.000)	0.935 (0.002)	0.973 (0.002)	0.982 (0.000)	0.983 (0.000)
	**Minimum**	0.984	0.985	0.932	0.971	0.982	0.984
	**Maximum**	0.985	0.985	0.938	0.976	0.982	0.982
**F1**							
	**Mean (SD)**	0.529 (0.005)	0.533 (0.006)	0.425 (0.008)	0.527 (0.014)	0.538 (0.004)	0.563 (0.003)
	**Minimum**	0.521	0.523	0.409	0.511	0.531	0.559
	**Maximum**	0.535	0.544	0.436	0.548	0.544	0.569
**Accuracy**							
	**Mean (SD)**	0.966 (0.000)	0.967 (0.000)	0.927 (0.002)	0.959 (0.002)	0.965 (0.000)	0.967 (0.000)
	**Minimum**	0.966	0.966	0.924	0.957	0.965	0.966
	**Maximum**	0.966	0.967	0.929	0.962	0.965	0.968

[a]ROS-ensemble: ensemble models using random over-sampling
[b]SMOTE-ensemble: ensemble models using Synthetic Minority Over-sampling Technique
